# Perfluorosulfonic Acid Membranes Modified with Polyaniline and Hydrothermally Treated for Potentiometric Sensor Arrays for the Analysis of Combination Drugs

**DOI:** 10.3390/membranes13030311

**Published:** 2023-03-08

**Authors:** Anna Parshina, Anastasia Yelnikova, Tatyana Kolganova, Tatyana Titova, Polina Yurova, Irina Stenina, Olga Bobreshova, Andrey Yaroslavtsev

**Affiliations:** 1 Department of Analytical Chemistry, Voronezh State University, 394018 Voronezh, Russia; 2 Kurnakov Institute of General and Inorganic Chemistry RAS, 119991 Moscow, Russia

**Keywords:** Donnan potential, potentiometric multisensory system, composite, PFSA, PANI, hydrothermal treatment, ion transport, sulfamethoxazole, trimethoprim, combination drug

## Abstract

A novel potentiometric multisensory system for the analysis of sulfamethoxazole and trimethoprim combination drugs was developed. The potentiometric sensors (Donnan potential (DP) was used as an analytical signal) with an inner reference solution were based on perfluorosulfonic acid (PFSA) membranes modified with polyaniline (PANI) by in situ oxidative polymerization. The order of the membrane treatment with precursor solutions and their concentrations was varied. Additionally, the PFSA/PANI composite membranes were hydrothermally treated at 120 °C. The influence of the preparation conditions and the composition of membranes on their sorption and transport properties was studied. We estimated the factors affecting the sensitivity of DP-sensors based on the PFSA/PANI composite membranes to ions of sulfamethoxazole and trimethoprim simultaneously presented in solutions. A developed multisensory system provided a simultaneous determination of two analytes in aqueous solutions without preliminary separation, derivatization, or probe treatment. The re-estimation of the calibration characteristics of the multisensory system did not show a statistically significant difference after a year of its use. The limits of detection of sulfamethoxazole and trimethoprim were 1.4 × 10^−6^ and 8.5 × 10^−8^ M, while the relative errors of their determination in the combination drug were 4 and 5% (at 5 and 6% relative standard deviation), respectively.

## 1. Introduction

The antibacterial activity of 4-aminobenzenesulfonic acid derivatives is enhanced in combination with trimethoprim (TMP). Combination drugs (medicines that include two or more active ingredients mixed in a single dosage form) based on 4-aminobenzenesulfonic acid derivatives and TMP are widely used in the treatment of respiratory, urinary, gastroenteric, and otolaryngologic infections; infections of the skin and soft tissues; meningitis; osteomyelitis; etc. [1,2,3].

To separate and then determine the components of combination drugs, high- and ultrahigh-performance liquid chromatography (LC) [4,5,6,7,8], microemulsion LC [9], hydrophilic interaction LC [10], and capillary electrophoresis [11,12] are used. The preliminary separation of active components makes the analysis difficult, time-consuming, expensive, and often polluting. Complex sample preparation can lead to a decrease in the analysis accuracy. The analysis of combination drugs without pre-separation and the derivatization of components can be performed using UV spectrophotometry with chemometrics [13,14,15]. A low peak resolution of analytes and an additivity rule violation are compensated for by multidimensional mathematical methods [14,15]. This approach is mainly used for the analysis of preparations containing two active ingredients, since an increase in the number of analytes increases the determination errors [13,14,15,16].

Electrochemical sensors have been developed for the selective determination of the components of drug combinations. A good resolution of oxidation peaks was achieved for some drug substances when voltammetric sensors based on a glassy carbon electrode, carbon paste electrode, or diamond electrode were used. For example, enalapril and hydrochlorothiazide can be determined using a carbon paste electrode based on functionalized carbon nanotubes [17]. The determination of captopril and hydrochlorothiazide was reported using sensors based on a carbon electrode modified with graphene and ferrocene [18] or with ionic liquid and Cu(OH)_2_ nanoparticles [19]. The work presented in [20] reports the simultaneous determination of pentoxifylline and paracetamol using a glassy carbon electrode modified with graphene nanoflakes. Sensors based on a glassy carbon electrode [21] or a diamond electrode doped with boron [22] were used for the determination of sulfametoxazole (SMX) and TMP. The disadvantage of the proposed systems was the need for a thorough pre-treatment of the SMX and TMP solutions, including their dissolution in alcohols, ultrasonic treatment or centrifugation followed by filtration, and the addition of buffer solutions, which were required to prevent poisoning of the sensory materials [21,22]. Moreover, the special mechanical and ultrasonic treatment of sensors [21] or their sequential treatment in isopropanol, deionized water, and sulfuric acid [22] was required before each measurement. The use of potentiometric sensors for the analysis of drug substances has been thoroughly reviewed elsewhere [23]. Potentiometric sensors with inner reference solutions and membranes based on poly(vinyl chloride) (PVC) and polystyrene, containing ion pairs of analytes with phosphotungstic heteropoly acid, have been reported for the simultaneous determination of ciprofloxacin and metronidazole [24] or tinidazole [25]. Two sensors and a reference electrode were used for the analysis [24,25]. The membrane composition of both sensors was the same, while the reference solutions were different: they included a 0.001 M solution of one of the analytes and 1 M KCl [24,25]. Solid-contact graphite electrodes with PVC membranes, containing ion pairs of fexofenadine–molybdate and cobalt–montelukast, were developed for the determination of fexofenadine hydrochloride and montelukast sodium in combination drugs [26]. Three sensors and a reference electrode were used to determine two analytes [26]. One sensor contained both ion pairs while two others contained an ionic pair of one of the analytes [26]. Carbon paste sensors based on molecularly imprinted polymers (MIPs) with imprints of alfuzosin or solifenacin provided their determination when they were both present in a solution [27]. The probe treatment of the preparations included a pH correction, particularly since the determination of each drug substances was performed at a different pH [26]. A solid contact sensor based on a PVC membrane containing a levetiracetam–tetraphenylborate ion pair showed good selectivity for palonosetron in the presence of inorganic ions and some anti-epileptic drugs [28]. The incorporation of calix[8]arene in the PVC membrane of a sensor with a liquid inner contact led to an increase in the selectivity for palonosetron in the presence of other components of drugs, including the degradation products of palonosetron [29]. The sensors’ lifetimes varied from 0.5 [24,25] to 1.5 [24,25,26,27,28,29] months. The benefits and drawbacks of the use of ionophores, in particular MIPs, in potentiometric sensors have been comprehensively reviewed elsewhere [30,31]. At the same time, a high selectivity of voltammetric and potentiometric sensors for components of combination drugs is not always achieved.

Small arrays of cross-sensitive (sensitive to more than one component), potentiometric (Donnan potential (DP) used as an analytical signal) sensors based on Nafion-type perfluorosulfonic acid (PFSA) membranes were developed for a multicomponent analysis of preparations based on 4-aminobenzenesulfonic acid derivatives [32,33]. PFSA membranes are attractive materials for the determination of biologically active analytes, due to their amphiphilic nature and the presence of a system of pores and channels, the size of which is about 5 and 1 nm in a hydrated state, respectively. Such membranes may be used as the sensing material of a sensor, which does not require additional protection from fouling [32,33,34], or as protection for the sensing material [35,36,37]. The sorption and transport properties of PFSA membranes may be changed by chemical modification (introduction of inorganic or organic particles (dopants)) or different treatment processes. Their properties depend on the combined effect of electrostatic, osmotic, and elastic forces on the membrane system of pores and channels containing a dopant [38]. Therefore, the permeability of modified membranes for ions of a different charge and size depends on the nature, concentration, and distribution of a dopant in the membranes [38]. A stable change in the system of pores and channels as the result of the PFSA membrane treatment at different temperatures and relative humidities is described in the literature as the “memory effect” [39,40,41]. This effect is due to a change in the membrane hydration and conformation transformation of polymeric chains, which are constrained at room temperature. This affects the distribution of sulfonic acid groups inside the material and the membrane pore size. At the same time, the effect of different treatment processes, especially hydrothermal treatments, on doped membranes has not been studied enough.

One of the strategies for improving PFSA membrane properties is doping/impregnating membranes with conductive polymers, e.g., PANI, which is cheap, simple to synthesize, and stable [42,43,44,45,46]. An optimization of the composition and preparation conditions of PFSA/PANI membranes should be realized to improve their characteristics and the performance of different devices based on them. Even a small amount of PANI reduces the diffusion through PFSA membranes, especially the gas permeability, and thereby protects cathode materials in direct methanol fuel cells [47,48] and hydrogen–air fuel cells [49,50] against poisoning. The high proton conductivity and water retention capacity of PFSA/PANI membranes at lower relative humidities is another important factor for their use in fuel cells [47,48,49,50]. Due to enhanced monovalent permselectivity, PFSA/PANI membranes can be used in electrodialysis water treatment [51] and hydrogen production via the Cu–Cl thermochemical cycle for platinum cathode protection [52]. A combination of the adhesive and conductive properties of PANI and the cation selectivity of PFSA membranes allows the use of PFSA/PANI composite membranes for the protection of electron conductors in electrochemical sensors [53,54,55].

It should be mentioned that PANI can complicate ionic transport through PFSA membrane due to the binding of sulfonic acid and amino groups and the blocking of membrane pores and channels [45]. A hydrothermal treatment (HT) can significantly change the properties of membranes [56], for example, leading to an increase in pore volume and water uptake and facilitating the packing of dopant polymer chains. On the other hand, an increase in the mobility of polymer chains during such treatment can lead to a change in the ion-exchange capacity of membranes modified by dopants with the opposite charge of functional groups (as in the case of PFSA and PANI) and an increase in ionic conductivity. Therefore, the possibility of an improvement of the sensitivity of potentiometric DP-sensors to SMX^−^ anions and TMP^+^ cations using a hydrothermal treatment (HT) of PFSA/PANI membranes was studied in this work. HT-treated PFSA/PANI membranes could exhibit an enhanced sorption ability for ions with aromatic and different acid–base properties due to an increase in the availability of reactive centers.

The aim of this work was the development of a potentiometric multisensory system with DP-sensors based on PFSA membranes, containing PANI and hydrothermally treated, for the analysis of SMX and TMP combination drugs.

## 2. Materials and Methods

### 2.1. Materials and Reagents

Commercial PFSA membranes (MF-4SC, Plastpolymer, Saint-Petersburg, Russia), aniline hydrochloride (>99%, Merck, Darmstadt, Germany), (NH_4_)_2_S_2_O_8_ (>98%, Sigma-Aldrich, Saint-Louis, MO, USA), HCl (special purity grade, Chimmed, Moscow, Russia), KCl (reagent grade, Chimmed, Moscow, Russia), sulfamethoxazole (4-amino-N-(5-methyl-3-isoxazolyl)benzenesulfonamide, 98%, Alfa Aesar, Ward Hill, MA, USA), trimethoprim (2,4-diamino-5-(3,4,5-trimethoxybenzyl)pyrimidine, ≥99%, Alfa Aesar, Ward Hill, MA, USA), Biseptol^®^ oral granules (Adamed Pharma, Czosnyw, Poland), and deionized water (resistance 18.2 MΩ, pH 5.41 ± 0.05, Simplicity^®^ Water Purification System, MerckMillipore, Darmstadt, Germany) were used.

### 2.2. Membrane Preparation

PFSA/PANI composite membranes were prepared by the in situ modification of commercial membranes according to a technique described elsewhere [32]. PANI was synthesized in the membrane pores via oxidative polymerization. The modified membranes containing the dopant (PANI), either along the whole length of the PFSA membrane or only up to half of its length, were fabricated. The homogeneously doped membranes were used for the membrane characterization (the investigation of the composition, structure, equilibrium, and transport properties), while the membranes with a gradient distribution of PANI were used for the DP-sensors. Some PFSA/PANI membranes were HT-treated. Both pristine and HT-treated commercial membranes were used as reference samples. They were designated as PFSA (pristine) and PFSA (HT), respectively.

Two series of composite membranes were prepared. They differed in the order of treatment with solutions of a monomer (aniline hydrochloride, ANI·HCl) and an oxidant ((NH_4_)_2_S_2_O_8_). The aniline hydrochloride concentration was 0.002, 0.005, or 0.010 M, while the (NH_4_)_2_S_2_O_8_ concentration was higher by 25%. In the first case (Method N1), the membranes were treated with the aniline hydrochloride solution (10 min), and then with the (NH_4_)_2_S_2_O_8_ solution (2 h). In the second case (Method N2), the treatment order was reversed. To manufacture membranes with a gradient distribution of PANI, the pristine membrane was immersed in a solution of aniline hydrochloride ((NH_4_)_2_S_2_O_8_) for only half its length. The membranes were then conditioned according to the standard procedure [49,50]. Some PFSA/PANI composite membranes were hydrothermally treated at 120 °C for 4 h using a Binder MKF115 climate chamber (Binder, Tuttlingen, Germany). Further, the monomer concentration, the preparation method, and the hydrothermal treatment were stated in the composite membrane labels, for example, PFSA/PANI (0.010 M, N1) and PFSA/PANI (0.010 M, N1, HT).

Since all the prepared membranes were in the H^+^ form, they were converted to the K^+^ form (for use as DP-sensors) by keeping them in 0.1 M KCl for 72 h and washing with deionized water. The membranes after long-term use in DP-sensors (up to 3 months) were regenerated in the same way. After a series of repeated measurements (about 100 measurements), the membranes were kept in a 0.1 M KCl solution for 30 min under constant stirring and stored in deionized water.

### 2.3. Preparation of Model Solutions and Pharmaceutical Solutions

Some characteristics of the analytes used (SMX and TMP) are listed in Table 1. The hydrophobicity is given for the molecular forms of the analytes (log *P* is the decimal logarithm of the octanol–water partition coefficient), as well as for the analytes simultaneously presented in molecular and charged forms (log *D*_pH=7_ is the decimal logarithm of the pH-adjusted octanol–water partition coefficient).

The model solutions for potentiometry contained SMX^−^ anions, TMP^+^ cations, and their molecular forms, SMX and TMP (hereafter, ions and molecules of TMP in equilibrium are designated as SMX^−^/SMX and TMP^+^/TMP). The concentrations of the components were in the range of 1.0 × 10^−5^–1.0 × 10^−3^ M. The pH values were in the range from 4.59 to 7.15; a buffer solution was not required.

The model solutions for spectrophotometry contained SMX^−^ anions and TMP molecules with concentrations ranging from 6.0 × 10^−6^ to 6.0 × 10^−5^ M, as well as an ammonium buffer solution to obtain a pH = 10.0.

To prepare 1 L of the Biseptol^®^ drug solution, one tablet was dissolved in deionized water. The solution was then filtrated to remove insoluble components. The prepared solution contained SMX^−^/SMX and TMP^+^/TMP, as well as poly(vinyl alcohol), the concentration of which was insignificant. The composition of the Biseptol^®^ preparation (per 1 tablet) and the pharmaceutical solution is given in Table 2. The pharmaceutical solution was used as received for the potentiometric analysis (pH = 5.371 ± 0.010), while for the spectrophotometric analysis, it was diluted by 10 times (pH = 10.0, ammonium buffer solution).

### 2.4. Experiment and Data-Processing Procedures

The sensor properties were investigated for the membranes in the K^+^ form, while the sorption and transport properties were investigated in the H^+^ and K^+^ forms.

The membrane water uptake was measured in the temperature range of 20–200 °C using a Netzsch-TG 209 F1 thermal balance (Netzsch, Selb, Germany).

The ion-exchange capacity (IEC) of membranes was determined by direct titration using 0.010 M NaOH. The IEC values were calculated per 1 g of a swollen membrane.

The equilibrium concentration of TMP^+^ cations in the membrane, as well as their sorption dynamics during the first 5 h after immersion of the membrane in the K^+^ form into the analyte solution, were evaluated by considering the change in the analyte concentration in the outer solution using a Shimadzu UV-1800 spectrometer (Shimadzu, Kyoto, Japan). The measurements were performed in quartz cells with an absorbance path length of 10 mm. The absorbance at a wavelength of 271 nm (*A*_271_) was measured in aqueous TMP solutions containing a sodium acetate buffer solution (pH = 4.0). Equation (1) was used as a calibration equation in the range of TMP concentrations (*c*_TMP_) from 2 × 10^−5^ to 2 × 10^−4^ M. The analyte concentration in the membrane was calculated per 1 g of a swollen membrane.

(1) $$A_{271}=5.7\times 10^{3}\times c_{\mathrm{TMP}}$$

The conductivity measurements were performed using an Elins Z500 PRO impedance meter (Elins, Zelenograd, Russia) in potentiostatic mode, with a frequency range of 10^−2^ × 10^6^ Hz. The measurements were performed using graphite paper/membrane/graphite paper symmetric cells with an electrode active surface area of ~1 cm^2^ in deionized water in the temperature range of 25–50 °C. To determine the membrane resistance at each temperature, the Nyquist plots were extrapolated to the Z′ axis.

The H^+^/K^+^ mutual diffusion coefficients and diffusion permeabilities were studied in a two-chamber cell separated by a membrane for 0.1 M KCl/0.1 M HCl and 0.1 M KCl/H_2_O solutions using an Ekonix-Expert 001 pH meter and an Ekonix-Expert 002 conductometer (Ekonix-Expert, Moscow, Russia), respectively.

A DP-sensor included the same components as a traditional potentiometric liquid-filled sensor: an inner reference electrode, an inner reference solution, and an ion-selective membrane (PFSA/PANI membrane). The electrochemical chain for the evaluation of the DP-sensor response was:

Ag|AgCl, sat. KCl|1 M KCl|PFSA/PANI membrane|test solution|sat. KCl, AgCl|Ag.(2)

Unlike the traditional sensor construction, an ion-selective membrane was not fixed in the DP-sensor and connected the reference solution and the test solution as a bridge. The distance between the reference and test solutions corresponded to the membrane length. This eliminated transmembrane transfer during measurements of the DP-sensor response and minimized the diffusion potential. The end of the modified part of the PFSA/PANI membrane was immersed into the test solution, while the end of the unmodified part was immersed into the reference solution (1 M KCl). The dopant absence in the part of the membrane contacted with the reference solution was necessary to eliminate a possible systematic error due to the dependence of the Donnan potential at this interface on the membrane composition. There was an intermediate part between the modified and unmodified parts of the PFSA/PANI membrane. It was no thicker than 0.5 cm, while the whole length of the membrane was 6 cm. The presence of the intermediate part did not introduce an error in the DP-sensor response, since the membrane ends were immersed into the test and reference solutions for ~0.3 cm, while the response time was no longer than 1 min. This was enough for the establishment of quasi-equilibration near the membrane interfaces with solutions, while the composition of the other part of the membrane was unchanged. Using chronopotentiometry, it was estimated that already after several seconds of contact with the test solution, the membrane’s response change did not exceed the response scatter upon experimental repetition (Figure S1 in Supplementary Materials). It was likely that quasi-equilibration near the membrane boundaries with the test and reference solutions was established, while the composition of the other membrane part did not change.

**Figure 1:**
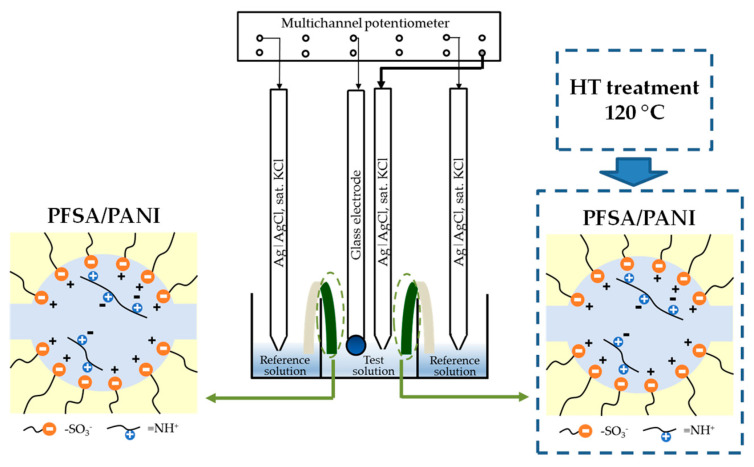
The cell scheme for the analysis of the Biseptol^®^.

The DP-sensor response was evaluated using a multisectional cell (Figure 1) described elsewhere [61,62]. The cell included one section for the test solution and eight sections for the reference solutions. This allowed measurements to be performed simultaneously for eight membranes of different compositions. The potential difference between AgCl electrodes (ESr-10103, Ekonix-Expert, Moscow, Russia) connected to an input for the reference electrode of the potentiometer and immersed into the test solution and AgCl electrodes connected to measurement inputs and immersed into the reference solution was subsequently measured using a multichannel potentiometer. Thus, the potential difference of several chains of type (2) was measured. An ES-10301/4 glass electrode (Ekonix-Expert, Moscow, Russia) was used for simultaneous pH measurements of the test solutions.

For the analysis of a drug containing SMX and TMP (Biseptol^®^), one section of the measurement cell was filled with the pharmaceutical solution and two sections were filled with the reference solution (Figure 1). The reference electrode and the glass electrode were immersed into the pharmaceutical solution. Two other reference electrodes were immersed into the sections with the reference solution. Two membranes connected the pharmaceutical solution and the reference solution (Figure 1). Membranes with optimal properties for the simultaneous determination of two analytes were chosen based on investigations of their sorption, transport, and sensor properties in model solutions.

To calibrate the DP-sensors in the multicomponent solutions, a regression analysis was used. The algorithm for experiment planning and the algorithm for the calculation of the equation coefficients using the least-squares method are thoroughly described elsewhere [32,33]. The calibration equations, which took into account the influence on the DP-sensor response (∆*ϕ_D_*, mV) of the negative decimal logarithm of the overall concentration of TMP molecules and TMP^+^ cations (pTMP), the negative decimal logarithm of the overall concentration of SMX^−^ anions and SMX molecules (pSMX), and the pH, were in the form of Equation (3). The calibration characteristics were established in the concentration range of the analytes, which was chosen according to the drug content. The linearity of the semilogarithmic concentration dependencies of the DP-sensor response in a wider concentration range of the analytes was not studied in this work. The significance of the calibration equation coefficients was evaluated by Student’s *t*-test. To control the adequacy of the calibration equations, the scatter (*ε*, mV) of the experimental response values relative to those predicted by the calibration equations was evaluated.

(3) $$\Delta \varphi_{D}=b_{0}+b_{1}\times \mathrm{pTMP}+b_{2}\times \mathrm{pH}+b_{3}\times \mathrm{pSMX},$$

where *b*_0_—the constant term of the calibration equation, mV, and *b_i_*—the sensitivity coefficient of the DP-sensor to the *i*-th component, mV/p*c*.

DP-sensors with minimal correlation between their responses according to the r-criterion were chosen for a multisensory system. To calculate the component concentrations in the pharmaceutical solution, a system of two calibration equations was solved using a matrix approach by substituting the measured values of the responses of two DP-sensors and their pH values. The limits of detection (LODs) of the analytes for a DP-sensor array were estimated by the “3σ” rule. For this purpose, a system of calibration equations for DP-sensors was solved by considering that the responses in a solution with the minimum detecting concentration differed from the responses in deionized water by no less than 3 values of the standard deviation. To prove the stability of the calibration characteristics of the DP-sensors, the characteristics were re-established after one year of their use.

A spectrophotometric method, described in [30], was used as a referent method for the analysis of the drug based on SMX and TMP. The best peak resolution on the UV-visible absorbance spectra for individual solutions of SMX (λ = 257 nm) and TMP (λ = 287 nm) was observed at pH = 10.0 (ammonium buffer). Nevertheless, a difference in the intensity of the analyte absorbance prevented the use of a traditional approach for their simultaneous determination. Therefore, a system of multidimensional regression equations was obtained to discriminate the responses. The calibration equations established in solutions containing both analytes at the wavelengths corresponding to the absorbance maxima of their individual solutions were in the form of Equation (4). The matrix approach was used to calculate the analyte concentrations.

(4) $$\{\begin{array}{c} A_{257}=16.8\times 10^{3}\times c_{\mathrm{SMX}}+2.2\times 10^{3}\times c_{\mathrm{TMP}},\\ A_{287}=2.9\times 10^{3}\times c_{\mathrm{SMX}}+6.9\times 10^{3}\times c_{\mathrm{TMP}}. \end{array}$$

## 3. Results and Discussion

### 3.1. Properties of Membranes

The PFSA polymer is capable of self-organization owing to the functionalization of hydrophobic macromolecules by hydrophilic groups. The structure of PFSA membranes is formed by the hydrophobic matrix and hydrophilic pores connected to each other with channels [38,40]. The synthesis of PANI by an in situ method occurred in the membrane pores where ions of the monomer (Ph-NH_3_^+^) and the oxidant (S_2_O_8_^2−^) were transferred. It was shown that the order of the membrane treatment with the monomer and oxidant solutions affected the distribution of PANI in the bulk of the modified membrane [32]. Ph-NH_3_^+^ cations easily and quickly exchanged with protons in the PFSA membrane. When the membrane in the Ph-NH_3_^+^ form was treated with the oxidant solution (Method N1-series samples), the dopant polymerization started in a thin surface layer of the membrane as S_2_O_8_^2−^ anions entered due to non-exchange sorption. The accumulation of PANI in the surface membrane layer blocked the further diffusion of S_2_O_8_^2−^ anions and aniline polymerization [32,50]. The concentration of S_2_O_8_^2−^ anions in the PFSA membranes that equilibrated with the oxidant solution (Method N2-series samples) was low. Therefore, with the further treatment of such membranes by the monomer solution, polymerization occurred only when a sufficient number of oxidized molecules of aniline were formed. This resulted in a more uniform distribution of PANI in the composite membrane [32,50]. The effectiveness of the PFSA membrane modification with PANI was proven by UV–visible and FTIR spectroscopies and scanning electron microscopy in [32].

All membranes containing PANI had lower IEC values than both the pristine and hydrothermally treated PFSA membranes (Table 3). An increase in the concentration of the monomer solution used to prepare the composite membranes led to a decrease in the IEC (Table 3). This was due to the formation of hydrogen bonds between N_X_H_Y_ fragments of PANI and the sulfonic acid groups of the membranes. The IEC of the N2-series membranes was slightly lower than the IEC of the N1-series membranes at the same monomer concentration (Table 3) due to the more uniform distribution of the dopant in the N2-series membranes. The concentration of the sulfonic acid groups in the membranes insignificantly changed under HT treatment (Table 3).

The introduction of a small amount of PANI into the membrane without further HT treatment slightly increased the membrane’s water uptake. However, it decreased with an increasing PANI concentration (for 0.005 and 0.010 M concentrations of ANI·HCl) (Table 3). A small amount of PANI in the membrane pores facilitated their expansion, but an increase in the dopant concentration led to the overlapping and cross-linking of the membrane pores due to the interaction of the dopant and membrane functional groups via hydrogen bonds. These effects were more pronounced for membranes in the H^+^ form. This might have been due to their higher hydration. Besides, when the membrane is in the H^+^ form, N_X_H_Y_ moieties of polyaniline are positively charged owing to protonation, which increases their affinity for the sulfonic acid groups of PFSA membranes. Under HT treatment, the water uptake increased for all membranes in the K^+^ form, as well as for the membranes in the H^+^ form, which did not contain the dopant and were prepared using the 0.002 M monomer solution (Table 3). A higher pressure of water vapor compared to the osmotic pressure inside a membrane promoted the sorption of an additional amount of water and the expansion of hydrophilic pores and channels. At the same time, the HT treatment, at a temperature higher than the glass transition temperature of the PFSA polymer, might be the reason for an increase in the mobility of its macromolecules and the macromolecules of PANI, as well as for an increased availability of their functional groups for the interaction with each other. This could increase the efficiency of the binding of protonated N_X_H_Y_ moieties to sulfonic acid groups when the membrane’s dopant concentration in the H^+^ form was high.

**Figure 2:**
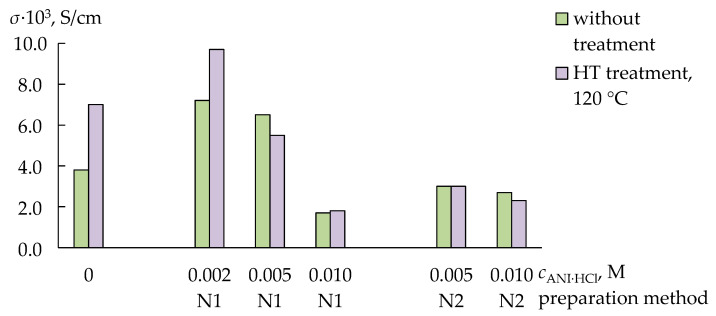
The conductivity (*σ*, S/cm) at 25 °C in deionized water of the PFSA/PANI and PFSA/PANI (HT) membranes.

**Figure 3:**
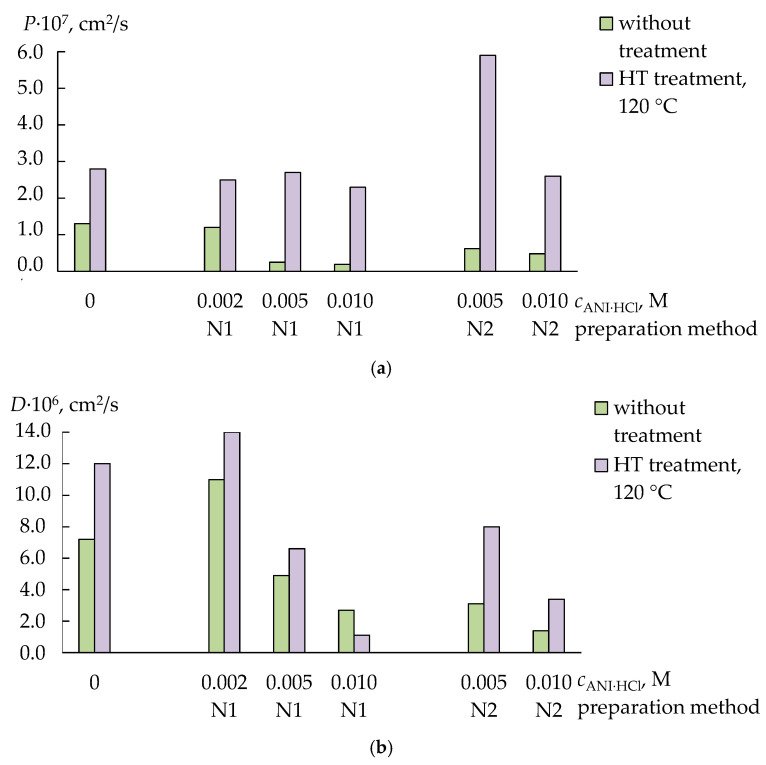
The diffusion permeability (*P*, 0.1 M KCl/H_2_O, cm^2^/s) (**a**) and H^+^/K^+^ mutual diffusion (*D*, 0.1 M HCl/0.1 M KCl, cm^2^/s) (**b**) of the PFSA/PANI and PFSA/PANI (HT) membranes.

The rate of ion transport through the composite membranes decreased with increasing PANI concentration (Figure 2 and Figure 3). This effect was more pronounced for the membranes firstly treated with the ANI·HCl solution and then with the oxidant (Figure 2 and Figure 3). The HT treatment of the pristine membranes and the membranes with the lowest PANI concentration led to an increase (by 1.3–1.8 times) in the ionic conductivity of these membranes in the K^+^ form (Figure 2). The HT treatment of the membranes prepared with the 0.005 or 0.010 M monomer solutions provided comparable or lower ionic conductivity than that of the membranes without the HT treatment (Figure 2). At the same time, the diffusion permeability of all membranes after the HT treatment significantly (by 2–12 times) increased (Figure 3a). A more pronounced increase in the diffusion permeability of the membrane in the K^+^ form, as well as the mutual diffusion of the H^+^ and K^+^ ions, was observed for the membranes of the N2-series (Figure 3a,b).

Thus, hydrothermally treated pristine membranes and membranes with the lowest PANI concentration had a more developed microstructure, which facilitated the transfer of counter- and co-ions. At the same time, the HT treatment of membranes with a higher PANI concentration led to the formation of larger hydrophilic clusters in the pore and channel system. This was evidenced by an increase in the water uptake and diffusion permeability when the IEC and ionic conductivity of these membranes were the same. An expansion of the hydrophilic pores and channels and, probably, the partial disentanglement of the macromolecules of the PFSA polymer under the HT treatment might be constrained because of hydrogen bonds between the functional groups of PANI and the membrane. Therefore, due to the presence in the membrane of the clusters, both containing the dopant and without it, the changes in the membrane morphology were non-uniform. If the surface layer was enriched with PANI (the N1-series membranes), the system of membrane pores and channels changed more uniformly under the HT treatment than in the membranes with pores both containing PANI and without it distributed all over the membrane (the N2-series membranes).

### 3.2. Cross-Sensitivity of the DP-Sensors

**Figure 4:**
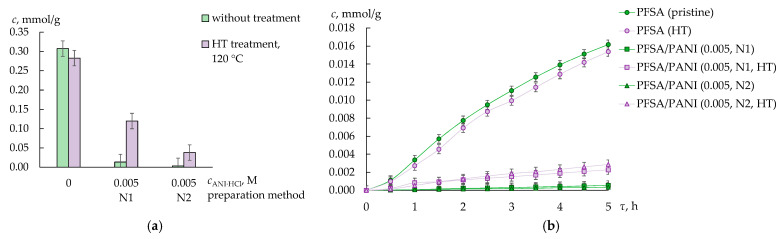
The changes in the concentration of TMP^+^ (*c*, mmol/g) in the PFSA/PANI membranes in contact with 1.0 × 10^−3^ M TMP solution (pH = 8.06 ± 0.03) for 72 h (**a**) and 2.0 × 10^−4^ M TMP solution (pH = 4.0, sodium acetate buffer) for 5 h (**b**).

Taking into account that PFSA membranes are cation-exchange membranes, DP-sensors based on them should have a high sensitivity to cations and an insignificant sensitivity to anions. However, such factors as the size and hydrophobicity of an analyte, as well as its non-exchange interactions with a membrane, affect its sorption and thereby change the sensitivity of DP-sensors to cations and anions. The size of TMP molecules and TMP^+^ cations is 0.703/0.750/1.228 nm (Table 1). This is comparable with the diameter of channels in the PFSA membrane (~1 nm [38]). At the same time, TMP molecules are hydrophobic (Table 1), while the presence of TMP^+^ cations minimizes the hydrophobicity of the analyte (Table 1). The amount of TMP^+^ cations sorbed by the pristine and hydrothermally treated PFSA membranes equilibrated with the 1.0 × 10^−3^ M analyte solution (pH = 8.06 ± 0.03) was lower by three times than the membrane IEC (Figure 4a). Taking into consideration that the size of the PFSA membrane pores is about 5 nm and that there are about eight to nine sulfonic acid groups in each pore, it seems that the presence of two to three TMP^+^ cations in the membrane pore corresponds with the maximum possible amount of the analyte in terms of the steric factor. Apart from the possibility of bulk cation transfer through the system of pores and channels, the availability of the TMP^+^ amino groups and the membrane sulfonic acid groups for electrostatic interactions or hydrogen bond formation should affect the concentration of the TMP^+^ cations in the membrane.

**Figure 5:**
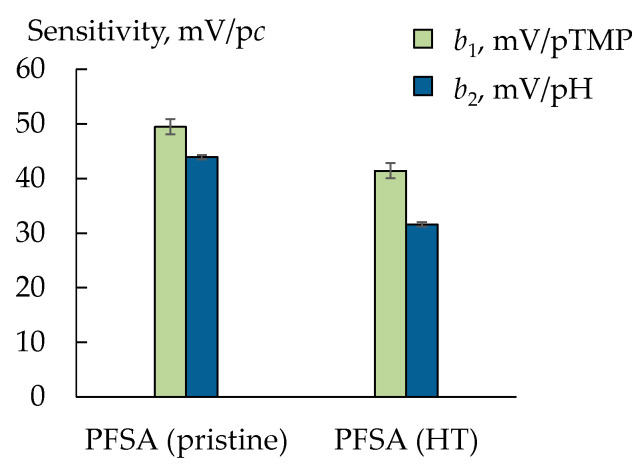
The sensitivity coefficients of DP-sensors based on PFSA membranes to TMP^+^/TMP and H_3_O^+^ (1.0 × 10^−5^–1.0 × 10^−3^ M, pH 6.14–8.06).

The sensitivity of DP-sensors to TMP^+^/TMP (if there were no other organic ions) reached 49.5 ± 1.4 and 41.4 ± 1.1 mV/p*c* for the PFSA (pristine) and PFSA (HT) membranes, respectively (Figure 5). At the same time, the sensitivity of the DP-sensor based on the pristine PFSA membrane to H_3_O^+^ ions was slightly lower (43.9 ± 0.4 mV/pH) than that to TMP^+^/TMP and decreased additionally after the HT treatment of the PFSA membrane (Figure 5). It seemed that some sulfonic acid groups were unavailable for the ion exchange as a result of the presence of the bulk analyte in the membrane. It should be mentioned that the presence of the TMP molecules in the solution also affected the DP-sensor response. The transfer of TMP molecules into the membrane should be followed by their protonation, since the pH of the intrapore solution is about 2 units lower than the pH of the outer solution. It was possible that the hydrophobic TMP molecules were adsorbed on the hydrophilic surface of the PFSA membrane (the water contact angle was 92.3 ± 0.5° [32]). That changed the conditions for sorption and ion exchange for the TMP^+^ and H_3_O^+^ cations.

**Figure 6:**
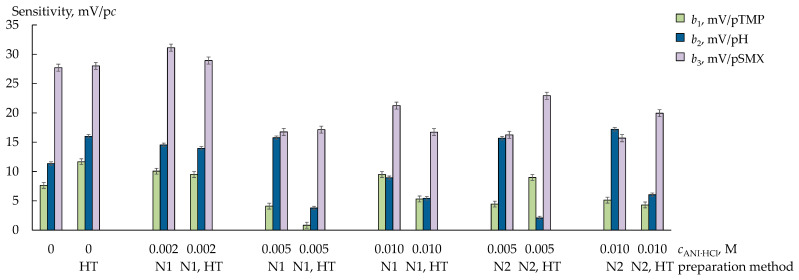
The sensitivity coefficients of DP-sensors based on the PFSA/PANI and PFSA/PANI (HT) membranes to SMX^−^/SMX, TMP^+^/TMP, and H_3_O^+^ (1.0 × 10^−5^–1.0 × 10^−3^ M, pH 4.59–7.15).

The sensitivity of the DP-sensors based on the PFSA (pristine) and PFSA (HT) membranes to TMP^+^/TMP and H_3_O^+^ decreased to 7.6–16.0 mV/p*c* if the solution contained SMX^−^ anions and SMX molecules (Figure 6). Meanwhile, the sensitivity of the DP-sensors to SMX^−^/SMX (27.7 ± 0.6, 28.0 ± 0.6 mV/p*c*) when the solution contained both organic analytes was comparable to the overall sensitivity of the DP-sensors to cations (Figure 6). This effect may be due to the affinity of the hydrophobic surface of the PFSA membrane to SMX^−^ anions and SMX molecules, which have hydrophobic properties, in particular under conditions when the charged fraction predominates in an aqueous solution (Table 1). It seemed that the SMX^−^ anions and SMX molecules orientated near the surface of the membrane owing to Van der Waals forces, preventing the transfer of TMP^+^/TMP and H_3_O^+^ into the membrane.

The presence of PANI in the PFSA membranes changed the conditions of the analyte sorption for a number of reasons. The extended PANI particles occupied a part of the pores and bound some sulfonic acid groups. This can create steric difficulties for the transfer of bulky analytes into a membrane and decrease the amount of sorption centers for TMP^+^ cations. At the same time, hydrophilic proton-acceptor N_X_H_Y_ fragments of PANI acted as sorption centers for SMX^−^ anions and facilitated some hydrophilization of the membrane surface (the water contact angles for the PFSA/PANI (0.005 M, N1) and PFSA/PANI (0.010 M, N1) membranes were 65.8 ± 0.4° and 71.5 ± 0.5° [32]). This should reduce the adsorption of analytes and promote their transfer into pores. Furthermore, stacking interactions of the analytes with π-conjugated moieties of PANI cannot be excluded.

The sensitivity of the DP-sensors based on the PFSA (HT), PFSA/PANI (0.002 M, N1), and PFSA/PANI (0.002 M, N1, HT) increased in the series of TMP^+^/TMP < H_3_O^+^ < SMX^-^/SMX in the multicomponent solutions (Figure 6). This tendency was the same as when the PFSA (pristine) membrane was used (Figure 6). However, these membranes provided a slight increase in the sensitivity of the DP-sensors to both the analytes and H_3_O^+^ ions in comparison to the PFSA (pristine) membrane (Figure 6). This is in accordance with data on the facilitation of the transfer of both cations and anions through these membranes (Figure 2 and Figure 3).

When the concentration of PANI in the membrane was higher (for the ANI·HCl concentrations of 0.005 or 0.001 M), the cross-sensitivity of the DP-sensors to the analytes and H_3_O^+^ ions significantly changed depending on the conditions of the membrane fabrication and treatment (Figure 6). The availability of sulfonic acid groups can be estimated by the change in the DP-sensor sensitivity to H_3_O^+^ ions in the presence of bulky organic analytes. A correlation between the change in the membrane selectivity to cations and the sensitivity of the DP-sensors based on them to H_3_O^+^ ions in solutions containing two organic analytes was observed. The DP-sensors based on the PFSA/PANI (0.005 M, N1) and PFSA/PANI (0.005–0.010 M, N2) membranes, which had a comparable or higher conductivity and a significantly lower diffusion permeability than the PFSA (pristine) membrane, were characterized by a high sensitivity to H_3_O^+^ ions. A decrease in the DP-sensor sensitivity to H_3_O^+^ ions was achieved for the PFSA/PANI (0.005–0.010 M, N1, HT) and PFSA/PANI (0.005–0.010 M, N2, HT) membranes, for which the ionic conductivity decreased while the diffusion permeability increased. It seemed that the facilitation of the non-selective transfer through the membrane upon HT treatment increased the concentration of the organic cations and anions in the membrane due to the increasing size of the intrapore space and the availability of sorption centers. This led to an exclusion of some sulfonic acid groups from the ion exchange.

The sensitivity of the DP-sensors based on the PFSA/PANI (0.005–0.010 M) membranes to SMX^−^/SMX was higher than that to TMP^+^/TMP. This was probably because the sorption conditions favored SMX^−^/SMX sorption more because of their smaller size (the volume of an SMX molecule is lower by two times than that of a TMP molecule (Table 1)) and an increase in the concentration of the anion-exchange and hydrophobic sorption centers in the membrane. At the same time, the DP-sensor sensitivity to TMP^+^/TMP and SMX^−^/SMX based on the membranes with a relatively high concentration of PANI was lower than that based on the membranes without PANI or with a small concentration of PANI. This evidenced the crucial influence of the steric factor on the concentration of organic analytes in the membrane. This was in good agreement with the fact that the equilibrium concentration of TMP^+^ cations in the membrane and the rate of their sorption decreased significantly when introducing PANI into the membrane, while the HT treatment otherwise led to an increase in the sorption ability of the PFSA/PANI membranes to TMP^+^ cations. This was more pronounced for the membranes of the N1-series (Figure 4).

### 3.3. Characteristics of the Multisensory Systems

The PFSA/PANI (0.005 M, N2) and PFSA/PANI (0.005 M, N2, HT) membranes were chosen for the simultaneous determination of SMX^−^/SMX and TMP^+^/TMP in aqueous solutions. They provided a high sensitivity to the analytes and a low correlation between the DP-sensor responses at the same time. The characteristics of the developed multisensory system, established in the model solutions with concentrations of both analytes ranging from 1.0 × 10^−5^ to 1.0 × 10^−3^ M (pH 4.59–7.15), are given in Table 4. The DP-sensors had a low variance in the response (*D*, mV^2^) and a small scatter of experimental response values relative to those predicted by the calibration equations (*ε*, mV). The LODs of SMX^−^/SMX and TMP^+^/TMP were 1.4 × 10^−6^ and 8.5 × 10^−8^ M, respectively. The relative errors of SMX^−^/SMX and TMP^+^/TMP determination in the model solutions were 2–11% (at 8–13% RSD) and 0.5–4% (at 9–16 RSD%), respectively (Table 4).

The re-estimation of the calibration characteristics of the DP-sensors chosen for the organization of a multisensory system did not show statistically significant differences after a year of their use if the storage and operation conditions were proper (Table 5).

The use of membranes in the K^+^ form was complicated by their fouling with ions of organic analytes. The affinity of the PFSA polymers to K^+^ ions was high; therefore, the equilibration of the membranes after a series of measurements with 0.1 M KCl provided their fast and complete regeneration. Besides, the spatial separation of the membrane interfaces with the test solution and the reference solutions of the DP-sensors minimized the diffusion of the components of the analyzed media into the membrane bulk. Electrostatic and hydrophobic interactions between the PANI and PFSA polymers prevented the leaching of the dopant from the membranes.

### 3.4. Application in Pharmaceutical Analysis

The determination of the active ingredients in the Biseptol^®^ tablets was performed using the multisensory system based on the PFSA/PANI (0.005 M, N2) and PFSA/PANI (0.005 M, N2, HT) membranes and the spectrophotometric method that was previously developed. The concentrations of SMX^−^/SMX and TMP^+^/TMP in the pharmaceutical solution (one tablet was dissolved in deionized water to prepare 1 L of the solution) measured by potentiometry were (3.8 ± 0.2) × 10^−4^ and (6.6 ± 0.4) × 10^−5^ M, respectively (Table 6). These values corresponded to the content of SMX and TMP in the preparation of 96 ± 5 and 19.0 ± 1.2 mg, respectively. The relative errors of the SMX and TMP determination in comparison to the content declared by the manufacturer were 4% (at 5% RSD) and 5% (at 6% RSD), respectively (Table 6). During the spectrophotometric analysis of the pharmaceutical solution (one tablet was dissolved in deionized water to prepare 1 L of the solution), the concentrations of SMX^−^ and TMP were found to be (3.87 ± 0.07) × 10^−5^ M (which corresponded to the content of 98.1 ± 1.7 mg in the preparation) and (6.71 ± 0.12) × 10^−6^ M (which corresponded to the content of 19.5 ± 0.3 mg in the preparation), respectively (Table 7). The relative errors of the results of the spectrophotometric determination of active ingredients in comparison to the content declared by the manufacturer were 1.9% for SMX^−^ (at RSD 2%) and 3% for TMP (at RSD 2%), respectively (Table 7).

The results of the potentiometric and spectrophotometric analyses were in good agreement. The relative errors of the SMX and TMP determination in the pharmaceutical preparation using the array of DP-sensors in comparison to the concentration found by the spectrophotometric technique were 3 and 2%, respectively (Table 6 and Table 7).

## 4. Conclusions

A potentiometric multisensory system for the simultaneous determination of sulfamethoxazole and trimethoprim in solutions of the combination drugs was developed. To organize cross-sensitive DP-sensors, the PFSA/PANI composite membranes were prepared by means of the reduction–oxidation polymerization of PANI inside the PFSA membrane. The content and distribution of PANI in the membrane depended on the order of the membrane treatment with the monomer and oxidant solutions and their concentration. The HT treatment of the PFSA/PANI membranes was performed to increase their sorption ability to bulky organic analytes with hydrophobic properties. The relations between the composition, preparation conditions of the PFSA/PANI membranes, and their sorption and transport properties were studied. The different influence of the HT treatment on the pristine and composite membranes was established and discussed. It was shown that the cross-sensitivity of DP-sensors based on the PFSA/PANI membranes to sulfamethoxazole and trimethoprim ions significantly depended on the steric factor and the non-exchange interactions. The multisensory system based on the PFSA/PANI membranes, which differed in the preparation conditions, provided a simultaneous determination of two analytes in aqueous solutions in the concentration range from 1.0 × 10^−5^ to 1.0 × 10^−3^ M (pH 4.59–7.15) without preliminary separation, derivatization, or probe treatment. The re-estimation of the calibration characteristics of the developed multisensory system did not show statistically significant differences after a year of its use. The LODs of sulfamethoxazole and trimethoprim were 1.4 × 10^−6^ and 8.5 × 10^−8^ M. The relative errors of the sulfamethoxazole and trimethoprim determination in the combination drug as compared to the content declared by the manufacturer were 4% (at 5% RSD) and 5% (at 6% RSD).

## Figures and Tables

**Table 1:** Some characteristics of SMX and TMP.

Characteristics	SMX	TMP
Chemical structure	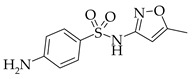	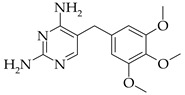
Size *, nm	0.526/0.587/1.031 [57]	0.703/0.750/1.228 [58]
Dissociation constant	pK_a1_ = 1.7, pK_a2_ = 5.6 [59]	pK_a_ = 7.12 [60]
Log *P*	0.89 [60]	0.91 [60]
Log *D*_pH=7_	0.11 [60]	0.03 [60]
Mole fractions at pH = 7	0.96 SMX^−^ 0.04 SMX	0.57 TMP^+^ 0.43 TMP

* The parameters (x/y/z) of 3D structure of the molecule.

**Table 2:** The composition of the Biseptol^®^ preparation and the pharmaceutical solution.

Ingredient	*m*, mg (1 Tablet)	*c*, M (1 Tablet/1 L Solution)
Active ingredients (soluble in water)		
Sulfamethoxazole	100.0	3.948 × 10^−4^
Trimethoprim	20.0	6.889 × 10^−5^
Excipients (insoluble in water)		
Potato starch	44.25	–
Talc powder	3.75	–
Magnesium stearate	1.25	–
Excipients (soluble in water)		
Poly(vinyl alcohol)	0.75	(1.5–37.5) × 10^−7^

**Table 3:** The IEC (±0.01 mmol/g) and water uptake (±0.5 wt%) of the PFSA/PANI membranes in H^+^ form and K^+^ form.

*c*_ANI·HCl_, M Preparation Method	IEC, mmol/g		Water Uptake, % in the H^+^ Form		Water Uptake, % in the K^+^ Form	
	Without Treatment	HT Treatment, 120 °C	Without Treatment	HT Treatment, 120 °C	Without Treatment	HT Treatment, 120 °C
-	0.77	0.76	16.0	17.9	12.7	17.4
0.002, N1	0.72	0.73	20.6	25.1	14.4	19.6
0.005, N1	0.71	0.71	19.1	18.0	14.0	16.2
0.010, N1	0.63	0.64	18.2	17.5	13.2	16.0
0.005, N2	0.69	0.70	20.7	22.0	13.7	21.4
0.010, N2	0.60	0.63	19.2	18.2	13.4	17.4

**Table 4:** The characteristics of the multisensory systems for the determination of SMX^−^/SMX and TMP^+^/TMP.

Characteristic		Membrane Composition	
		PFSA/PANI (0.005 M, N2)	PFSA/PANI (0.005 M, N2, HT)
*b*_0_ ± Δ*b*_0_, mV		−75 ± 9	−140 ± 5
*b*_1_ ± Δ*b*_1_, mV/pTMP		4.4 ± 0.5	9.0 ± 0.3
*b*_2_ ± Δ*b*_2_, mV/pH		−15.6 ± 0.3	−2.06 ± 0.14
*b*_3_ ± Δ*b*_3_, mV/pSMX		−16.3 ± 0.6	−22.9 ± 0.3
*ε*, mV		3	1.5
*D*, mV^2^		30	17
Response time, min		<1	
Working pH range		4.59–7.15	
Concentration range, M		1.0 × 10^−5^–1.0 × 10^−3^	
Stability, month		≥12	
LOD, M	SMX^−^/SMX	1.4 × 10^−6^	
	TMP^+^/TMP	8.5 × 10^−8^	
RSD, % (*n* = 4, *p* = 0.95)	SMX^−^/SMX	8–13	
	TMP^+^/TMP	9–16	
Relative error, %	SMX^−^/SMX	2–11	
	TMP^+^/TMP	0.5–4	

**Table 5:** The comparison of the calibration characteristics of the developed multisensory systems before and after their use for a year.

Membrane Composition	*b*_0_, mV		*b*_1_, mV/pSMX		*b*_2_, mV/pTMP		*b*_3_, mV/pH		*t*-Test, *f* = 8, *p* = 0.95	*F*-Test, *f*_1_ = 5, *f*_2_ = 3, *p* = 0.95
	*t*	*F*	*t*	*F*	*t*	*F*	*t*	*F*		
PFSA/PANI (0.005 M, N2)	0.54	1.42	1.89	1.21	0.60	1.40	0.64	1.31	2.31	8.91
PFSA/PANI (0.005 M, N2, HT)	0.74	1.03	0.45	1.24	0.81	1.14	0.34	1.08		

**Table 6:** The analysis of the Biseptol^®^ preparation using the multisensory system.

Membrane Composition	*c*, M (Pharmaceutical Solution *)		*c*, mg (Preparation)		RSD, % (*n* = 5, *p* = 0.95)		Relative Error **, %	
	SMX^−^ /SMX	TMP^+^ /TMP	SMX	TMP	SMX	TMP	SMX	TMP
PFSA/PANI (0.005 M, N2)	(3.8 ± 0.2)·10^−4^	(6.6 ± 0.4) × 10^−5^	96 ± 5	19.0 ± 1.2	5	6	4	5
PFSA/PANI (0.005 M, N2, HT)								

* One tablet was dissolved in deionized water to prepare 1 L of the solution, pH = 5.371 ± 0.010. ** The relative error was calculated by comparing to the content declared by the manufacturer.

**Table 7:** The analysis of the Biseptol^®^ preparation using the spectrophotometric technique.

*c*, M (Pharmaceutical Solution *)		*c*, mg (Preparation)		RSD, % (*n* = 6, *p* = 0.95)		Relative Error **, %	
SMX^−^	TMP	SMX	TMP	SMX	TMP	SMX	TMP
(3.87 ± 0.07)·10^−5^	(6.71 ± 0.12) × 10^−6^	98.1 ± 1.7	19.5 ± 0.3	2	2	1.9	3

* One tablet was dissolved in deionized water to prepare 1 L of the solution, pH = 10.0 (ammonium buffer). ** The relative error was calculated by comparing to the content declared by the manufacturer.

## Data Availability

Not applicable.

## Supplementary Materials

The following supporting information can be downloaded at: https://www.mdpi.com/article/10.3390/membranes13030311/s1, Figure S1: Chronopotentiometric curves of the responses of the DP-sensors based on PFSA membranes without PANI (a) and with PANI (b).
