# Supplementary material for: Perfluorosulfonic Acid Membranes Modified with Polyaniline and Hydrothermally Treated for Potentiometric Sensor Arrays for the Analysis of Combination Drugs
Source: Membranes (Basel). 2023 Mar 8;13(3):311. doi: 10.3390/membranes13030311 (PMC10058550; doi:10.3390/membranes13030311)
Supplement: Supplementary file 1 [file membranes-13-00311-s001.zip › membranes-2249708-supplementary.pdf]

membranes

Supplementary Materials

Perfluorosulfonic Acid Membranes Modified with Polyaniline and Hydrothermally Treated for Potentiometric Sensor Arrays for the Analysis of Combination Drugs

Anna Parshina [1,*], Anastasia Yelnikova [1], Tatyana Kolganova [1], Tatyana Titova [2], Polina Yurova [2], Irina Stenina [2], Olga Bobreshova [1] and Andrey Yaroslavtsev [2]

[1] Department of Analytical Chemistry, Voronezh State University, Voronezh 394018, Russia
[2] Kurnakov Institute of General and Inorganic Chemistry RAS, Moscow 119991, Russia

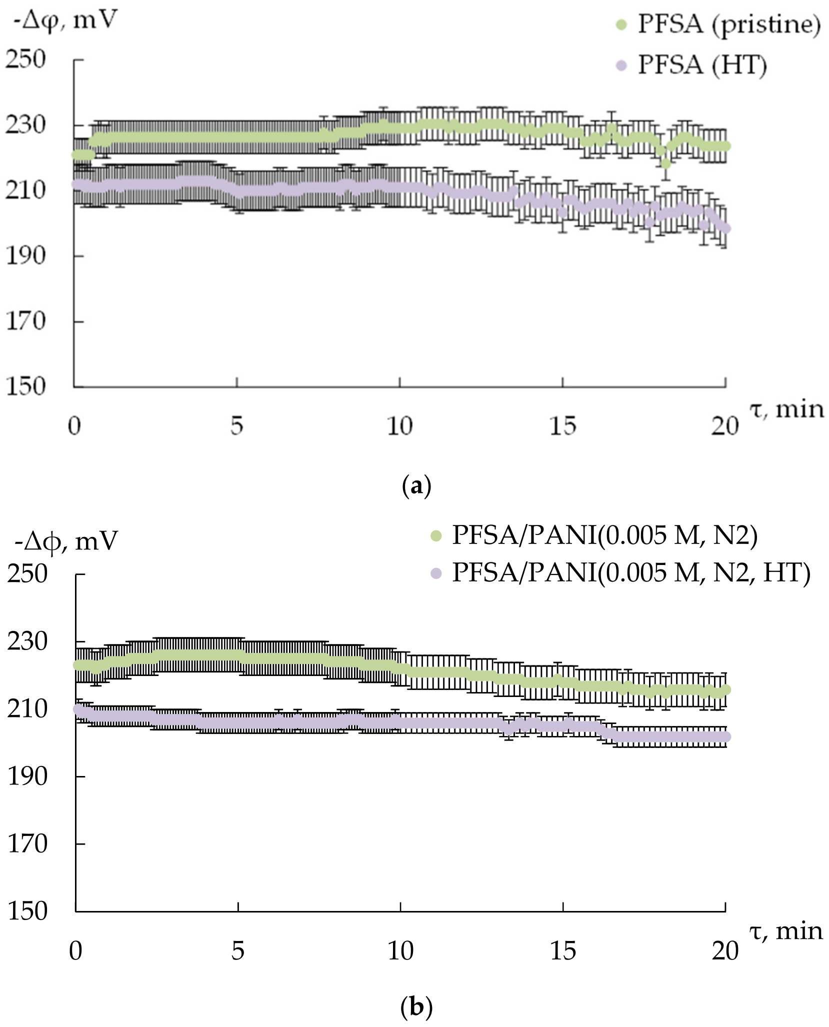

Figure S1. Chronopotentiometric curves of the responses of DP-sensors based on PFSA membranes without PANI (**a**) and with PANI (**b**).
